# Wiring the retinal circuits activated by light during early development

**DOI:** 10.1186/1749-8104-9-3

**Published:** 2014-02-13

**Authors:** Gabriel E Bertolesi, Carrie L Hehr, Sarah McFarlane

**Affiliations:** 1 Department of Cell Biology and Anatomy, Hotchkiss Brain Institute, University of Calgary, 3330 Hospital Dr. NW, Health Sciences Building, Room 2164, Calgary AB T2N4N1, Canada

**Keywords:** c-fos, Melanopsin, Eye, Vision, Circadian rhythm, Xenopus

## Abstract

**Background:**

Light information is sorted by neuronal circuits to generate image-forming (IF) (interpretation and tracking of visual objects and patterns) and non-image-forming (NIF) tasks. Among the NIF tasks, photic entrainment of circadian rhythms, the pupillary light reflex, and sleep are all associated with physiological responses, mediated mainly by a small group of melanopsin-expressing retinal ganglion cells (mRGCs). Using *Xenopus laevis* as a model system, and analyzing the *c-fos* expression induced by light as a surrogate marker of neural activity, we aimed to establish the developmental time at which the cells participating in both systems come on-line in the retina.

**Results:**

We found that the peripheral retina contains 80% of the two melanopsin-expressing cell types we identified in *Xenopus*: melanopsin-expressing horizontal cells (mHCs; *opn4m*+/*opn4x*+/Prox1+) and mRGCs (2.7% of the total RGCs; *opn4m*+/*opn4x*+/Pax6+/Isl1), in a ratio of 6:1. Only mRGCs induced *c-fos* expression in response to light. Dopaminergic (tyrosine hydroxylase-positive; TH+) amacrine cells (ACs) may be part of the melanopsin-mediated circuit, as shown by preferential *c-fos* induction by blue light. In the central retina, two cell types in the inner nuclear layer (INL) showed light-mediated induction of *c-fos* expression [(On-bipolar cells (Otx2+/Isl1+), and a sub-population of ACs (Pax6−/Isl1−)], as well as two RGC sub-populations (Isl1+/Pax6+ and Isl1+/Pax6−). Melanopsin and opsin expression turned on a day before the point at which *c-fos* expression could first be activated by light (Stage 37/38), in cells of both the classic vision circuit, and those that participate in the retinal component of the NIF circuit. Key to the classic vision circuit is that the component cells engage from the beginning as functional ‘unit circuits’ of two to three cells in the INL for every RGC, with subsequent growth of the vision circuit occurring by the wiring in of more units.

**Conclusions:**

We identified melanopsin-expressing cells and specific cell types in the INL and the RGC layer which induce *c-fos* expression in response to light, and we determined the developmental time when they become active. We suggest an initial formulation of retinal circuits corresponding to the classic vision pathway and melanopsin-mediated circuits to which they may contribute.

## Background

Since vertebrates arose some 500 million years ago, the requirements of their photoreception have evolved to a complex assembly of neuronal circuits, the mammalian retina. In mammals, the retina is the only structure that detects light, sorting information for two fundamentally different light-dependent processes. The image-forming (IF) circuit interprets and tracks visual objects and patterns, while the non-image-forming (NIF) circuits encode the environmental illumination for responses such as circadian rhythms, pupil size, and sleep [1]. Although the cells that make up the two systems coexist and interconnect in adult retinas, how and when they become functional and integrate into neuronal networks at early development times has received little attention [2].

Three retinal photoreceptive cell types process light information in mammals: cones and rod photoreceptors, and a small number of intrinsically photosensitive, melanopsin-expressing retinal ganglion cells (mRGCs) [3-5]. The rods and cones reside in the outer nuclear layer (ONL), and constitute the first cells of the conventional IF retinothalamocortical primary pathway. In a simplified IF circuit, photoreceptors connect with two types of inner nuclear layer (INL) cells, the bipolar cells (BCs) and the horizontal cells (HCs). In turn, bipolar and amacrine cells (ACs) synapse with the classic retinal ganglion cells (RGCs), which project their axons to the areas of the brain responsible for vision [6].

Expression of the melanopsin (*opn4*) gene confers photosensitivity to the minor population of mRGCs, and *opn4* mouse mutants have impaired NIF responses [4,7]. In mammals, mRGCs deliver features of ambient light. Most mRGCs extend axons to the suprachiasmatic nucleus (SCN), the olivary pretectal nucleus, and the activating neurons in the ventrolateral preoptic area, to regulate NIF tasks such as the photic entrainment of circadian rhythms, the pupillary light reflex, and sleep responses, respectively [7-10]. A minor projection to conventional visual centers is also present in the adult brain [11,12]. In mammals, neuronal circuits that induce IF and NIF responses are interconnected in the adult retina. Light evokes in mRGCs both the melanopsin-based response and synaptically mediated signals that originate from photoreceptor activation [13,14]. Indeed, rods sense dim light levels and work through mRGCs to entrain the endogenous circadian rhythm [15,16]. The time at which the interconnection between the rod/cone pathway and mRGCs occurs during development is unknown.

In contrast to mammals, lower vertebrates possess cell types outside of the eye which are photosensitive, and these are located in a variety of sites within and outside of the central nervous system. For example, the pineal gland contains photosensitive neurons [17-19]. The presence of additional light-sensitive organs in lower vertebrates does not release the eye from a role in NIF tasks. Indeed, isolated retinas cultured from *Xenopus laevis* exhibit robust circadian rhythms that can be reset by light [20]. Moreover, melanopsin (*opn4*) and other circadian genes are expressed in the retina of lower vertebrates [21-23]. Of note, mammals have a single melanopsin gene, *opn4m*, whereas non-mammalian vertebrates have two, the mammalian-like *opn4m* and the *Xenopus*-like *opn4x*[24-26].

Here, we aimed to identify the point during early vertebrate development at which the light-sensitive, neuronal retina networks become functional. We used as a model system the well-characterized retina of the *X. laevis* tadpole, in which the neuronal circuits are readily accessible for light activation at early developmental times. We describe the cells present in the early light-activated retinal circuits, and the time at which they become integrated into functional circuits. To do so, we assessed *c-fos* induction in response to light. *c-fos* is an early immediate gene that is induced by post-synaptic neurons, and that identifies light-activated retinal and brain cells involved in IF and NIF tasks [27-31].

We observed that in the retina of *Xenopus* both melanopsin-expressing cells and those that participate in the classic visual pathway become active at the same developmental time (Stage 37/38), concurrent with the establishment of retinal layers and synaptic connections, and innervation of brain targets by RGC axons. Further, we found that cells in the central retina engage as a complete functional circuit, with participating cells able to communicate from the outset with their downstream partners. This initial circuit then grows during early development by the addition of ‘circuit units’, comprising two to three cells in the INL for every RGC.

## Results

### Light induces *c-fos* expression in the INL and the RGC layer as early as Stage 37/38

To identify the cells involved in light-sensitive circuits in the retina, we chose a developmental time point at which the retinal neuronal circuits underlying IF and NIF responses are established and functional. The development of the cell types involved in the IF circuitry of *X. laevis* has been studied [32-34]. Visual inputs begin to innervate the major midbrain target, the optic tectum, at developmental Stage 37/38, and functional synapses are present at Stage 41 [32-34]. Analysis of behavioral tasks, such as visual avoidance, confirms a functional visual system at Stage 44 [35]. With regards to the NIF system, a functional circadian system is present within the pineal gland at Stage 26, and within the retina by Stage 41, as measured by the induction of melatonin release [18]. Thus, we chose Stage 42 for our analyses.

**Figure 1:**
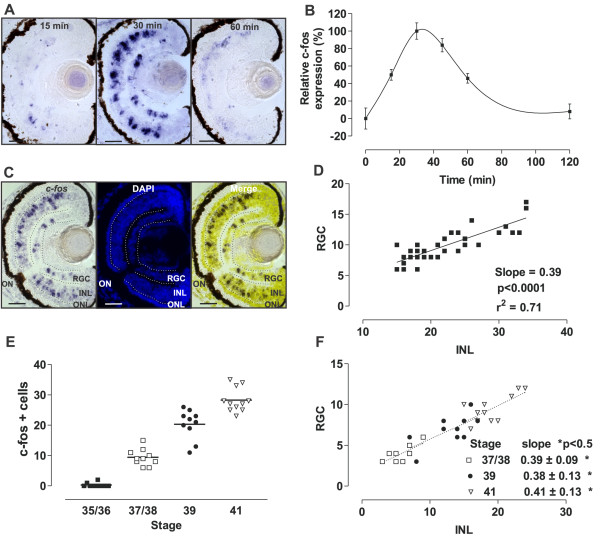
**Light induces *c-fos *expression in the inner nuclear layer (INL) and retinal ganglion cell (RGC) layer as early as Stage 37/38. (A)***c-fos in situ* hybridization of transverse sections from Stage 42 tadpole eyes that developed in the dark and were exposed to light (2500 lux) for the indicated times. **(B)** Graph of the integral optical density of *c-fos* in the eye (mean ± SEM; n = 8 eyes) relative to that measured after 0 (0%) or 30 (100%) minutes of light exposure. **(C)***c-fos in situ* hybridization (left), DAPI staining (middle), and corresponding merged picture (right) of a representative central section used to quantify *c-fos +* cells in the INL and the RGC layer. **(D**) Correlation between the numbers of *c-fos*-expressing cells in the INL and the RGC layer. Data for each central retina quantified are represented by a dot (n = 33). The linear regression and the coefficient of regression are indicated. **(E)** Embryos at different stages of development were exposed to light (2500 lux, 30 minutes) and the number of *c-fos +* cells in a section from the central retina quantified. Differences between all stages analyzed were statistically significant (*P* < 0.05; one-way ANOVA, Bonferroni’s multiple comparisons test). Line indicates the mean. **(F)** Correlation between the numbers of *c-fos +* cells in the INL and the RGC layer. The slope for each group is indicated (*P* value for each linear regression from Stages 35/36 and older are statistically significant; *P* < 0.05; there were no significant differences in the slopes between different stages). ONL, Outer nuclear layer; ON, Optic nerve. Scale bar = 50 μm.

*c-fos* is an immediate early gene activated in neuronal circuits in response to trans-synaptic stimulus [28,31]. Thus, we used *c-fos* expression as a marker of neuronal activity, analyzing the expression induced by light. Taking advantage of the fact that *c-fos* is virtually undetectable in the inner retina in the absence of light [30], we set *Xenopus* tadpoles to develop in the dark from Stage 24 (before retinal circuits have formed and the pineal gland releases melatonin) until testing for light-induced expression of *c-fos* at Stage 42. By restricting our analysis to tadpoles exposed to light for the first time, we also avoided complications with retinal adaptation and/or light-induced apoptosis, which alter *c-fos* expression [36]. Initially, we examined the time course of *c-fos* induction in the eye by exposing the embryos to a continuous source of white light (2500 lux). *c-fos* mRNA in the central sections of the eye was detected by *in situ* hybridization shortly after light exposure, and reached a maximum at 30 minutes. Subsequently, the levels declined and were almost undetectable by 2 hours (Figure 1A,B). Sense RNA probes produced no labeling above background (data not shown).

*c-fos* + cells localize to both the INL and RGC layer, and are absent from the ONL. We quantified the number of *c-fos +* cells in the RGC layer and INL in central sections from different eyes (n = 33). DAPI staining after *in situ* hybridization facilitated the counting and identification of each *c-fos +* cell (Figure 1C). An analysis of all sections allowed us to identify a high degree of correlation in the number of *c-fos +* cells present in the INL with respect to those in the RGC layer. The data obtained had a good fit with a slope of 0.39 ± 0.04 (mean ± standard deviation (SD)) (Figure 1D), indicating that for every cell activated in the RGC layer there were two to three *c-fos +* cells in the INL (expected slopes of 0.5 and 0.33, respectively).

We next analyzed the expression of *c-fos* over the period of synaptic wiring in the developing eye. Embryos developed in the dark, and were exposed to light (2500 lux) for 30 minutes at different stages over the period of circuit formation. *c-fos* + cells in the central retina were not detected until Stage 37/38 (Figure 1E), when positive cells appeared simultaneously in the INL and the RGC layer. After this, the number of *c-fos +* cells increased steadily (mean ± SD (*n*); Stage 37/38: 9.4 ± 2.7 (10); Stage 39: 20.3 ± 4.9 (10); Stage 41: 28.3 ± 4.0 (11)) (Figure 1E). These results indicate that in the central retina the light-induced response is generated in cells as early as Stage 37/38, the time when BC and HC dendrites synapse with photoreceptors [37] and the first RGC axons reach the tectum. Interestingly, the ratio of the number of *c-fos +* cells in the INL to those seen in the RGC layer remained constant at all stages analyzed (Figure 1F), suggesting that the turning on of synapses between photoreceptors and INL-activated cells, and between INL cells and RGCs, occurs almost simultaneously (Stage 37/38). The number of *c-fos +* cells continued to increase throughout development, arguing for the recruitment of additional circuits, but always, with the fixed ratio of two to three cells in the INL to a single RGC.

Together, these data suggest that in the central retina, the first exposure to light rapidly induces *c-fos* mRNA in cells located both in the INL and the RGC layer. Further, *c-fos* can be used reliably to identify newly forming circuits as early as Stage 37/38, and recruitment of cells to the IF circuits occurs in functional units of two to three cells in the INL for every RGC.

### On-BCs and two sub-populations of RGCs turn on *c-fos* in response to light in the central retina

**Figure 2:**
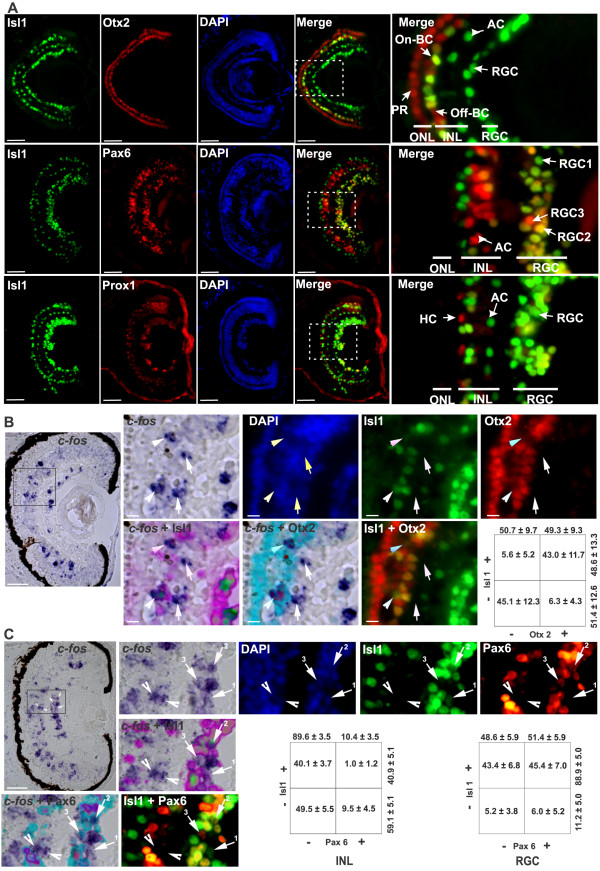
**Distinct cell types induce *c-fos *expression in response to light. (A)** Transverse Stage 42 retinal sections co-immunolabeled for Isl1 (green), and either Otx2 (top), Pax6 (middle), or Prox1 (bottom) (red). Higher magnification of a merged picture (right column) and DAPI staining (middle column; blue). The Isl1 antibody recognized On-BCs and ACs in the inner nuclear layer (INL), and RGCs in the RGC layer. Otx2 stained PRs in the outer nuclear layer (ONL) and On-BCs (Otx+/Isl1+) and Off-BCs (Otx+ / Isl1−) cells. Pax6 identified ACs in the INL, and RGCs. Three populations of cells in the RGC layer were defined by Pax6 and Isl1 expression: Isl1+ / Pax6− (RGC1; green), Isl1+ / Pax6+ (RGC2; yellow) and Isl1− / Pax6+ (RGC3; red). Prox1+ HCs were in the outer region of the INL. **(B)** Light induced *c-fos* in On-BCs. *c-fos* mRNA in central retinal sections of dark-reared Stage 42 embryos exposed to light (2500 lux) for 30 minutes, followed by immunohistochemistry against Isl1 (green) or Otx2 (red), and by DAPI staining (blue). A higher magnification of the region is indicated, and the corresponding merges are shown. Two *c-fos+* / Otx2+ / Isl1+ (arrowheads) and two *c-fos+* / Otx2− / Isl1− (arrows) cells are indicated. The percentage of *c-fos +* cells in central retinal sections expressing the corresponding markers (mean ± SD; n = 10 retinas) in the INL is shown in tabular form. **(C)** Two sub-populations of RGCs expressed *c-fos. In situ* hybridization against *c-fos,* immunohistochemistry against Isl1 (green) or Pax6 (red), and DAPI staining (blue). Two *c-fos+* / Pax6− / Isl1− cells (arrowhead) in the INL, and three *c-fos +* cells (arrows) corresponding to RGC1 (Isl1+ / Pax6−), RGC2 (Isl1+ / Pax6+), and RGC3 (Isl1− / Pax6+) are indicated. Scale bar = 50 μm for lower, and 10 μm for higher, magnifications.

In order to characterize the light-responsive circuits, we needed to identify the different cell types that induce c-*fos* after light exposure. To do so, we followed *in situ* hybridization for *c-fos* with immunohistochemistry for several transcription factors (Otx2, Isl1, Pax6, and Prox1) that are markers for specific retinal cell types (Figure 2A). Transcription factors were chosen because the nuclear localization of the proteins, determined by immunohistochemistry, was easy to compare with mRNA expression as detected by *in situ* hybridization. To identify On-BCs and Off-BCs, we used antibodies against Otx2 and Isl1. Members of the Otx family of transcription factors (Otx2 and Otx5) play a crucial role in the cell fate specification of *Xenopus* photoreceptors and BCs [38], whereas Isl1 is restricted to cholinergic ACs and On-BCs in the INL and to RGCs [39] (Figure 2A). We found that in the INL, On-BCs were co-immunoreactive with Otx2 and Isl1 antibodies, while Off-BCs were immunoreactive only for Otx2 (Figure 2A). ACs and RGCs, whose cell bodies localize to the inner portion of the INL and the RGC layer, respectively, were immunoreactive for Pax6 and Isl1 [40] (Figure 2A). Finally, HCs were recognizable by the expression of Prox1, which participates in HC formation [41]. HC nuclei were easily recognized as they localize in the outer INL and were Prox1+/ Isl1− (Figure 2A).

To understand what proportion of the different cell types turn on *c-fos* in response to a first exposure to light, we determined the percentage of specific cell types that were *c-fos +* with respect to the total number of immunoreactive cells for each marker, analyzed in central retinal sections from Stage 42 embryos. Calbindin and rhodopsin immunostaining (cone and rod markers, respectively) confirmed the lack of *c-fos +* cells in the ONL (data not shown). In the INL, only a small proportion (12.2 ± 3.2%; mean ± SD) of the total number of Otx2+ On-BCs were *c-fos* + (88 ± 18 Otx2 immuoreactive cells/section; n = 10). Similar results were obtained with Isl1 in the INL (20.3 ± 4.7% Isl1+ / *c-fos+*; 70 ± 10 Isl1+ cells/section; n = 19), while there were almost no Pax6+ ACs (6.5 ± 1.3%; 73.4 ± 11 cells/section; n = 9) or Prox1+ HCs (3.9 ± 2.8%; 21.9 ± 5.6 cells/section; n = 10) expressing *c-fos*. Finally, in the RGC layer, similar numbers of Pax6+ cells (13.0 ± 2.6%; 58.9 ± 11.4 cells/section; n = 9) and Isl1+ cells (13.9 ± 4.7%; 91.8 ± 15.9 cells/section; n = 19) induced *c-fos*. These results indicate that at Stage 42 approximately 10 to 15% of BCs and RGCs turn on *c-fos* in response to an initial 30 minute light exposure, whereas first light does not induce *c-fos* expression in HCs and photoreceptors.

Because several markers were not expressed exclusively in a single cell type, we next used a combination of markers to identify *c-fos +* cells more specifically. To identify On BCs, we counted the number of *c-fos +* cells that were immunoreactive with Otx2 and Isl1 antibodies in the INL. Approximately half of the *c-fos +* cells in the INL were Otx2 immunoreactive, and these were found next to the outer plexiform layer (OPL), while the other (Otx2−) half resided in the inner part of the INL (277 *c-fos+*, 138 Otx2+ and 139 Isl1+ cells counted; n = 10 sections) (Figure 2B). Using both markers, we found that 43 ± 11% of the *c-fos +* INL cells in each section were Otx2+ / Isl1+, probably corresponding to On BCs (n = 10 sections; n = 119 cells). Based on their location, and the lack of Otx2 immunoreactivity, we suggest that the remaining *c-fos +* cells (n = 122) in the INL were ACs. These cells (122 cells) were Isl1− (Figure 2B) and thus were unlikely to be cholinergic ACs, which express Isl1 [39]. They also did not express the AC marker Pax6 (89.6 ± 3.5% of the *c-fos +* cells in the INL were Pax6−; n = 19; n = 367 *c-fos +* cells counted) (Figure 2C, INL).

We next quantified the expression of the population of *c-fos +* cells in the INL and the RGC layer using Isl1 and/or Pax6 markers. In the INL, approximately half of the cells (40.1 ± 3.7%; n = 367 *c-fos +* cells counted) were Isl1+ / Pax6− and were localized to the outer part of the INL, corresponding to the previously described On BCs (Otx+ / Isl1+ / Pax6−), while the other half (49.5 ± 5.5%) were negative for both markers analyzed (Isl1− / Pax6−). Interestingly, in the RGC layer, three distinct populations of cells were recognized by double staining for Isl1 and Pax6. Almost all the *c-fos +* cells in the RGC layer were Isl1+ (88.9 ± 5.0%; n = 20; n = 332 cells counted) (Figure 2C), and of these, approximately half expressed Pax6 (RGC2: *c-fos+* / Isl1+ / Pax6+) (Figure 2C). As displaced cell types normally form a small proportion of the cells located in the RGC layer, the two main populations of *c-fos* + cells (Isll+ / Pax6− and Isll+ / Pax6+) were unlikely to be displaced ACs. There was a small number of *c-fos +* cells in the RGC layer that did not express Isl1 but expressed Pax6, with only 20 such cells found in the 20 central retinal sections analyzed (RGC3: *c-fos+* / Isll− / Pax6+; n = 332 *c-fos +* cells counted). Further analyses (see below) indicated that this population corresponds to the minor melanopsin-expressing mRGCs, and not to displaced ACs.

These results show that in the central retina of Stage 42 tadpoles, light induces *c-fos* expression in On BCs (Otx2+ / Isl1+) and a sub-population of Pax6− / Isl1− cells, possibly ACs, which sit close to the inner plexiform layer (IPL). In concert, two similarly sized populations of RGCs (Isl1+ / Pax6+ and Isl1+ / Pax6−) express *c-fos*.

### Expression of melanopsins in the retina of *Xenopus* tadpoles

**Figure 3:**
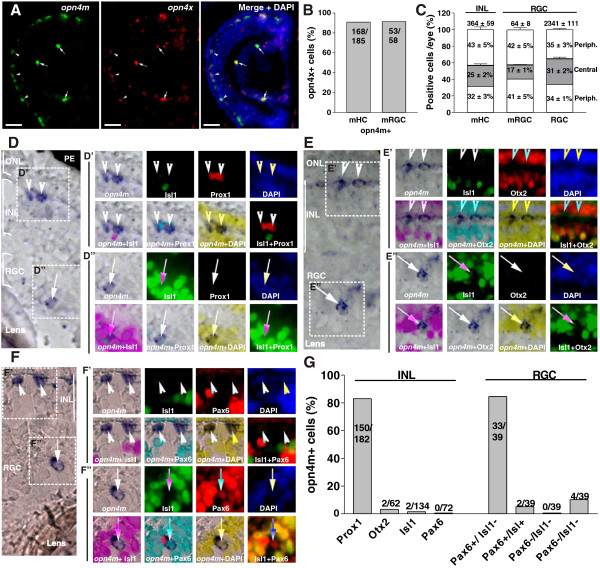
**Expression of melanopsin (*opn4m *and *opn4x*) in the *Xenopus *tadpole retina. (A)** Double *in situ* hybridization against *opn4m* and *opn4x* in a transverse section of the retina of a Stage 42 tadpole. Merged photograph with DAPI staining is shown (right). Double-positive cells in the outer nuclear layer (ONL) (melanopsin-expressing horizontal cells (mHCs); arrowheads) and in the retinal ganglion cell (RGC) layer (melanopsin-expressing retinal ganglion cells (mRGCs); arrows) are indicated. Scale bar = 100 μm. **(B)** Quantification of the number of *opn4x* + cells that also expressed *opn4m*, shown as percentage. The number of cells counted is indicated. **(C)** Distribution of mHCs and mRGCs counted in consecutive sections throughout the whole eye, divided into two peripheral domains and one central domain. The percentage of cells located in each domain, and the total numbers of cells counted (mean ± SD; n = 3 eyes), are indicated. The total number of RGCs counted (based on DAPI + nuclei), and their distribution, is also shown. **(D-F)***In situ* hybridization identified *opn4m* + cells in the outer segment of the INL (**D′**-**F′**, arrowheads) and in the RGC layer (**D′**-**F′**, arrows). *In situ* hybridization was followed by immunohistochemistry against Isl1 (green) or Prox1 (red) **(D′ and D′′)**, Isl1 and Otx2 (red) **(E′ and E′′)**, or Isl1 and Pax6 (red) **(F′ and F′′)**. Nuclei stained with DAPI (blue) and merged photograph of the corresponding images are presented. **(G)** The percentage of cells double-labeled for *opn4m* and the indicated marker in the INL and the RGC layer, as well as the number of cells counted, are shown.

We next investigated the emergence of the intrinsically photosensitive system of the retina. Melanopsin (*opn4m*) is the gene that confers photosensitivity to mammalian mRGCs (also called intrinsically photosensitive RGCs) and plays a central role in NIF tasks [3,5,8,9]. Thus, we used melanopsin expression to mark cells in the Stage 42 tadpole retina that were likely to be intrinsically photosensitive. First, we assessed the expression of mammalian-like *opn4m* and *Xenopus*-like *opn4x* by *in situ* hybridization [21,22,24]. In mammalian retina, *opn4m* mRNA is restricted to mRGCs [3,42], whereas in lower vertebrates, *opn4m* and/or *opn4x* are expressed in different species and different retinal cell types, including in HCs, BCs, photoreceptors, and mRGCs [21,24,43-47]. We found in the embryonic *Xenopus* retina that *opn4m* and *opn4x* cells were localized to the outer half of the INL, where the somas of HCs reside (hereafter called melanopsin-expressing horizontal cells; mHCs), and in the RGC layer (mRGCs) (Figure 3A). The double *in situ* hybridization revealed significant co-localization, with more than 90% of the cells expressing both melanopsins (Figure 3B). Consequently, we restricted additional analysis to *opn4m*. These data were in agreement with previous studies in chicken [24], although differences have been observed in salmon [48] and developing chicks [45].

We next counted the total number of mHCs and mRGCs in the whole eye in consecutive transverse sections (for mHCs an average of 24 sections containing the INL and ONL, and for mRGCs an average of 21 sections containing the RGC layer) and analyzed their distribution (central versus peripheral). mHCs were located mainly in the dorsal retina (arrowheads; Figure 3C) and were six times more abundant than the mRGCs (mean ± SD: mHCs: 364 ± 59 versus mRGCs: 64 ± 8; n = 6 eyes). As in mammals, only a handful of RGCs (2.7 ± 0.2%; 2341 ± 111 DAPI + cells in the RGC layer; n = 6 eyes) expressed *opn4m* (arrows, Figure 3A,B). This percentage is almost identical to that estimated in rodents [3,49]. Only 17 ± 1% of the mRGCs were localized to the central retina (seven central sections with the middle section containing the optic nerve), with the remaining 83% found in the periphery, even though the numbers of (DAPI+) cells in the RGC layer was similar in each third of the retina analyzed (Figure 3C).

The majority of *opn4m*-expressing cells in the INL corresponded to HCs, in that they were Prox1+ / Isl1− (82%, n = 182 cells) (Figure 3D,D**′**,G), and did not express cell markers for On-BCs (3.2% Otx2+ / Isl1+; n = 62 cells) (Figure 3E,E**′**,G), or ACs (4.1% Isl1+, n = 72 cells; 0% Pax6+, n = 72 cells) (Figure 3F,F**′**,G). Thus, *Xenopus* was similar to chicken and fish in having a population of mHCs [22,24,43-45]. In teleost fish, mHCs are intrinsically photosensitive [50], raising the possibility that this is also true of mHCs in *Xenopus*. The majority of *opn4m +* RGCs (84.6% n = 39) were Pax6+ Isl1− (Figure 3F,F**′′**,G), corresponding to the minor population of *c-fos* + RGCs (RGC3) discussed above.

In summary, melanopsin + cells were distributed mainly in the retinal periphery, and included both mHCs (Prox1+ / Otx2− / Isl1− / Pax6−) and mRGCs (Pax6+ / Isl1−). Importantly, these data support the idea that *c-fos* induction in the central retina is largely restricted to cells that participate in the classic vision-related, melanopsin-independent circuits.

### mRGCs turn on *c-fos* in response to light as early as Stage 37/38, whereas mHCs do not

**Figure 4:**
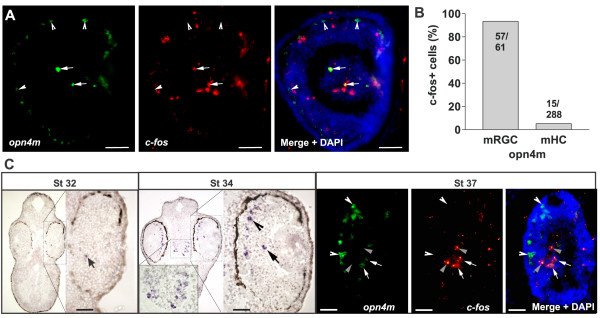
**Light induces *c-fos *expression in melanopsin-expressing retinal ganglion cell (mRGCs) but not melanopsin-expressing horizontal cells (mHCs) as early as Stage 37/38. (A)** Double *in situ* hybridization against *opn4m* and *c-fos* on a transverse retinal section from a Stage 42 embryo. Merged image with DAPI staining is also shown. **(B)** Quantification of the number of *c-fos* + cells that also express *opn4m* in the RGC layer (mRGCs) and in the INL (mHCs) expressed as percentages. The number of cells counted is indicated. **(C)***In situ* hybridization against *opn4m* (Stage 32 and 33/34) (left panels) and double *in situ* hybridization against *opn4m* and *c-fos* (Stage 37/38) (right panels). Left: Transverse sections through the peripheral eye (higher magnification Stage 32 and 33/34) and the brain (Stage 33/34) are shown. mHCs (*opn4m+ / c-fos−*; arrowhead), mRGCs (*opn4m+ / c-fos+*; arrow) and RGCs (*opn4m− / c-fos+*; gray arrowhead) are indicated. Scale bar = 50 μm.

We investigated whether *c-fos* induction could be used to monitor the activation of the embryonic NIF retinal circuit. We assessed by double *in situ* hybridization the expression of *c-fos* by retinal *opn4m* + cells after 30 minutes of light stimulation (2500 lux). Although almost all of the mRGCs that expressed *opn4m* also expressed *c-fos* after light induction (93%; n = 61 cells), the *opn4m +* mHCs did not (5.3%; n = 288 cells) (Figure 4A,B). Similar results were observed with *opn4x* + cells (data not shown). These results indicate that photosensitive cells in the outer retina, such as mHCs, and rods and cones of the classic visual circuit, do not express *c-fos* after a first exposure to light. By contrast, cells of the inner retina, including On BCs, putative ACs (Pax6− / Isl1−), RGCs and mRGCs turn on *c-fos* after light stimulation.

To determine whether melanopsin-expressing cells become active over the same developmental time window as the INL cells and RGCs of the classic light-activated retinal circuit (Stage 37/38), we examined when *opn4m* + expression begins. *opn4m +* expression was first detected in a few retinal cells at Stage 32 (Figure 4C), prior to lamination of the retina. By Stage 33/34, melanopsin + cell numbers had increased not only in the eye, where both cell types could be recognized by their laminar position, but also in the brain (Figure 4C). Similar to the IF circuit, however, light did not induce *c-fos* in *opn4m* + cells until Stage 37/38, and induced *c-fos* in mRGCs but not in mHCs (Figure 4C).

### A small, peripherally localized population of TH + cells express *c-fos* in response to light

**Figure 5:**
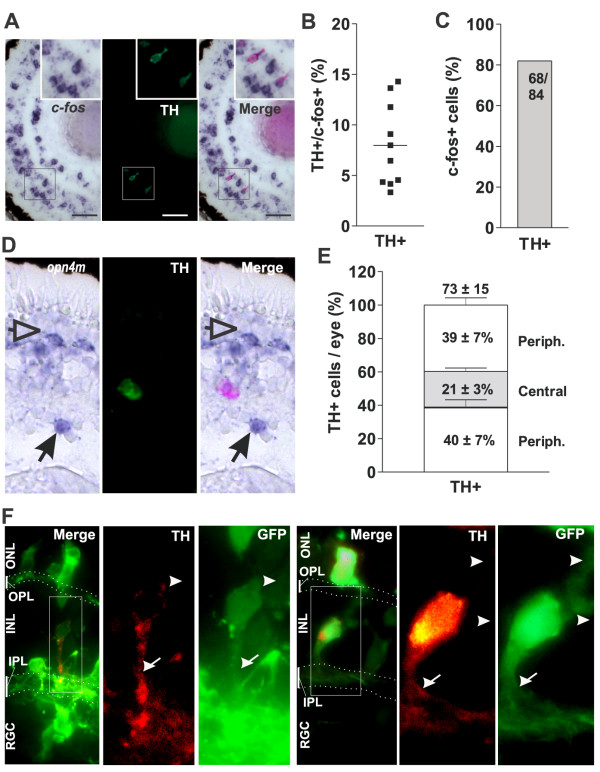
**Amacrine tyrosine hydroxylase (TH)-positive cells express *c-fos *in response to light and localize mainly in the peripheral retina. (A)***In situ* hybridization against *c-fos* of a central retinal section from a Stage 42 tadpole exposed to light (2500 lux, 30 minutes) followed by immunohistochemistry against TH. Insets show higher magnification of a *c-fos+* / TH + cell. **(B)** Percentage of TH + cells relative to the total number of *c-fos +* cells in each central retinal section is indicated by a dot; the horizontal line represents the mean (n = 10). **(C)** Percentage of TH + cells exhibiting *c-fos* induction by light. The number of cells counted is indicated. **(D)** Transverse section of the eye from a Stage 42 tadpole showing a representative *in situ* hybridization label for *opn4m* and immunohistochemistry results for TH. Merge image is shown in the right panel. mHC (arrowheads) and mRGC (arrows) are indicated. **(E)** Distribution of TH + cells counted in consecutive sections throughout the whole eye, divided into two peripheral areas and one central area. The percentage of the cells located in each region and the total numbers of cells counted (mean ± SD; n = 3 eyes) are indicated. **(F)** Morphology of the TH + cells. Stage 28 embryos were electroporated with a GFP construct. At Stage 42, Double-positive cells (TH + and GFP+) were revealed by immunohistochemistry. Examples of two cells are shown, with the merged image shown on the left, and the higher magnification of the TH + (red) and GFP (green) cells shown in the right panels. Neurite extensions (TH+ / GFP+; arrow) oriented to the inner plexiform layer (IPL), and neurites (TH− / GFP+; arrowhead) oriented to the outer plexiform layer (OPL), are indicated. The IPL and OPL are shown by dots. INL, inner nuclear layer; ONL, outer nuclear layer; RGC, retinal ganglion cell layer.

To complete our characterization of the cells participating in the central light-activated circuits, we attempted to identify the population of light-induced, *c-fos*-expressing ACs that express neither Isl1 nor Pax6. One possibility was that these were dopaminergic, tyrosine hydroxylase (TH)-positive ACs that previously were shown to turn on *c-fos* in response to light [51,52]. However, immunolabeling after *c-fos in situ* hybridization indicates that in Stage 42 central retina only a few *c-fos +* cells in the INL were TH + (8.0 ± 4.1% ; n = 10; n = 237 *c-fos +* cells counted) (Figure 5A,B), clearly not accounting for the much larger number of central Isl1− / Pax6− / *c-fos* + ACs. Indeed, the total number of TH + cells in the whole eye of a Stage 42 tadpole was 73 ± 15 (n = 3 eyes). Of these, more than 80% showed strong *c-fos* induction in response to light (Figure 5C). Further, *in situ* hybridization against *opn4m* followed by TH immunohistochemistry showed that TH + cells were often found in close proximity to *opn4m +* cells in the same or adjacent sections (Figure 5D), and possessed a similar distribution throughout the eye, with only 21% of the TH + cells located in the central retina (compare mRGCs in Figures 3C and 5E).

Two populations of TH + cells have been described in the adult *X. laevis* retina. The interplexiform ACs extend short processes towards the OPL and a neurite to the IPL, while the more classic ACs extend a process to the IPL [53]. In order to characterize the morphology of the TH + ACs, we electroporated a construct encoding GFP into the Stage 28 eye primordium [54], and used GFP to assess the morphology of TH+/GFP + cells at Stage 42 (Figure 5F). Most of the cells possessed a single TH+ / GFP + neurite that branched in the IPL (Figure 5F, arrow), and a few small GFP+ / TH− neurites oriented to the OPL (Figure 5F, arrowhead).

In summary, these results show that TH + cells are polarized cells that express *c-fos* in response to light, but these results cannot account for the significantly larger population of *c-fos +* cells of the inner region of the central INL. The number, localization, and distribution of TH + cells and mRGCs correlate, raising the possibility that they participate in a common circuit.

### Differential induction of retinal *c-fos* expression with different light colors

Next, we investigated if *c-fos* expression could be triggered differentially by light with different wavelengths. Similar to the adult *Xenopus* retina [37,55], we found a comparable proportion of rods and cones in the Stage 42 retina. Immunolabeling revealed that of the DAPI + cells in the ONL, 54.4% were rhodopsin + rods and 45.6% were calbindin + cones (mean ± SD: 68.3 ± 4.3 rhodopsin + cells/section (54.4%) versus 57.2 ± 6.4 calbindin + cells/section (45.6%); n = 6 sections). Rods and cones in *Xenopus* were shown previously to have distinct wavelength sensitivities [37]. Almost all rods (97%) were sensitive to green light (λ_max_ = 523), with less than 3% absorbing in the blue region of the spectrum (λ_max_ = 445), whereas the vast majority of cones (86%) were sensitive to long wavelengths (red; λ_max_ = 611 nm) [37]. By contrast, cells expressing melanopsin (mRGCs or heterologous systems overexpressing melanopsin) showed a maximum response in the blue region (~λ_max_ = 480 nm) [43,56,57]. Based on the differential wavelength sensitivities for photoreceptors and melanopsin-expressing cells, we hypothesized that circuits downstream of photoreceptors should mainly be activated by red or green light, and those that involve melanopsin-expressing cells should mainly be activated by blue light.

**Figure 6:**
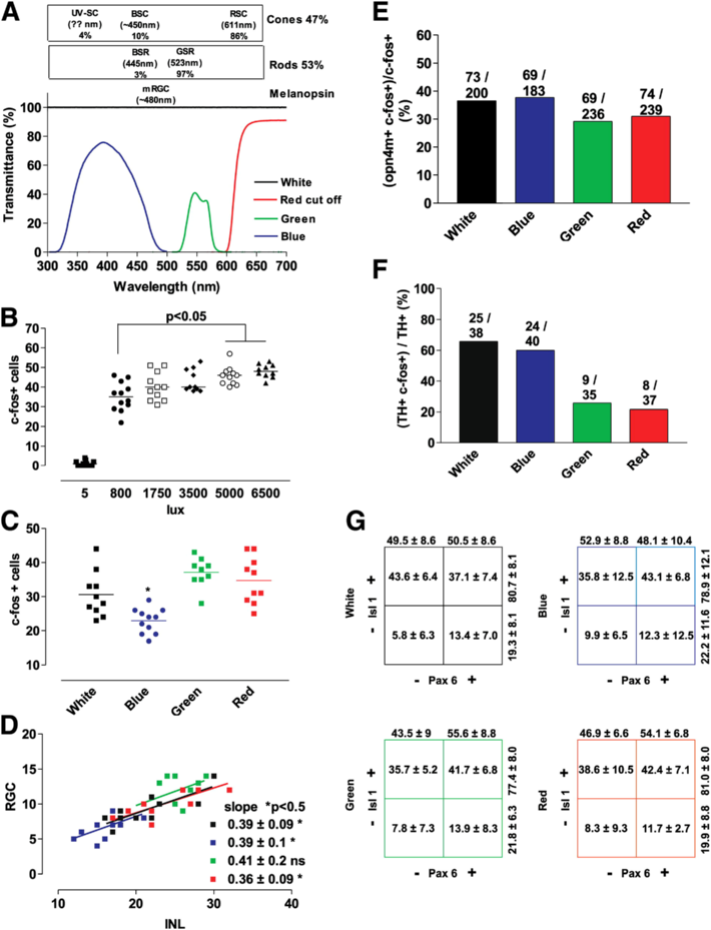
**Differential *c-fos *induction mediated by light colors. (A)** Spectrum of transmittance for the blue, green, and red cut-off filters used to determine color dependence of *c-fos* activation. Also indicated are the different types of photoreceptors and their proportions in the retina of adult *Xenopus laevis*, as well as the maximum wavelength sensitivity for the corresponding opsins [37]. Ultraviolet (UV)-sensitive cones (UV-SCs), blue-sensitive cones (BSCs), and red-sensitive cones (RSCs) are present, as well as blue-sensitve rods (BSRs) and green-sensitive rods (GSRs). **(B, C)** Quantification of the number of *c-fos +* cells in a central retina section of a Stage 42 tadpole exposed to the indicated light intensities **(B)** or colors **(C)** for 30 minutes. The horizontal lines indicate the means. Statistics: One-way ANOVA plus Bonferroni’s multiple comparison tests.* *P* < 0.05. **(D)** Correlation between the numbers of *c-fos*-expressing cells in the inner nuclear layer (INL) and retinal ganglion cell (RGC) layer after colored light exposure. Data for each central retina quantified are represented by a dot (n ≥ 10). The slope of the linear regression and the statistical analysis of the coefficient of regression are indicated. **(E)** Percentage of activated mRGC (*opn4m + c-fos+*) with respect to the total number of *c-fos +* responsive cells in the RGC layer counted in successive sections throughout the whole eye as determined by double *in situ* hybridization. The average number of *c-fos* + mRGCs/eye is indicated (n = 2). **(F)** Percentage of TH + cells exhibiting *c-fos* expression after induction with different light colors for 30 minutes. The number of cells counted is indicated. **(G)** The number of *c-fos +* cells expressing Pax6 and/or Isl1 in the RGC layer in the central retina (expressed as percentages; mean ± SD; n = 10) after exposure to different light colors are represented in tabular form.

In order to conduct these experiments, Stage 42 tadpoles were exposed to 30 minutes of light passed through specific filters that allowed the embryos to be exposed selectively to red (cut-off filter <600 nm), green (filter λ range 520 to 587 nm; λ_max_ = 547 nm) or blue (filter λ range 315 to 500 nm; λ_max_ = 400 nm) light (Figure 6A). Of note, different wavelengths are absorbed differentially by the water in which the embryos swim. Red-orange wavelengths (600 to 700 nm) are better absorbed than the medium yellow-green (500 to 600 nm) wavelengths, with the short visible wavelengths absorbed most poorly (violet blue; 400 to 500 nm) [58]. We were concerned as to whether the filtered light could generate sufficient energy (intensity) to induce *c-fos* expression. We found that the number of *c-fos* responsive cells increased dramatically from the dark (5 lux) condition, to an 800 lux light intensity, and increased only slightly with higher light intensities (800 lux versus 5000 lux; *P* < 0.5) (Figure 6B). Thus, to ensure that the filtered light passing through 1 cm depth of water generated an estimated intensity of more than 800 lux, we set the white light source to produce 3,500 lux at the surface of the dish [58].

We first compared *c-fos* responsiveness to different light wavelengths of cells in the central retina, where few mRGCs reside. The aim of these experiments was to use color to investigate whether the PR and melanopsin-dependent circuits are functionally distinct from one another at early stages of circuit development. If blue light activates only the melanopsin-dependent system, only mRGCs (and possibly TH + ACs) should be activated in response to light. Fewer *c-fos* + cells were present in central sections from embryos exposed to blue light compared with those exposed to white, green, and red light (Figure 6C). The decrease, however, was not large enough to fit with a scenario where only melanopsin + cells are activated with our blue light paradigm, as mRGCs are a minor population of the *c-fos* + cells in the central retina. Instead, it appears that the photoreceptors of the IF circuit do respond to blue light, but less effectively than to white, red, or green light (Figure 6C). In support of this idea, there was no change in the INL:RGC layer ratio of *c-fos +* cells with the different light colors (linear regression with similar slopes; approximately 0.39) (Figure 6D), suggesting that although fewer circuits were activated with blue light, the downstream cell types involved in the central retina circuits were similarly independent of the light color that triggered the activation. Interestingly, red and green light appeared equally as effective at turning on *c-fos* expression in mRGCs (*opn4m+*) as blue light, as determined by double *in situ* hybridization (Figure 6E). These data may reflect a paradigm whereby melanopsin is differentially activated by different wavelengths of light [57], whereas the *c-fos* readout is binary, and is the same for cells with either low or high neural activity. However, the results could also indicate a contribution by the PR vision circuit to mRGC neural activity. In lower vertebrates, PRs are interconnected by gap junctions [59], and thus activation of a distinct spectral sensitive PR sub-population would be quickly transmitted to the greater PR population. Intriguingly, light colors induced differential activation of TH + ACs, in that the proportion that turned on *c-fos* was similar after activation with white and blue light, and significantly greater than that observed with green and red light (Figure 6F), suggesting that TH + cells may contribute to a melanopsin-activated circuit.

Finally, we analyzed if one or other of the two similarly sized RGC populations that induced *c-fos* in response to white light (RGC1: Isl1+ / Pax6− and RGC2: Isl1+ / Pax6+) were preferentially activated by either rods or cones. Although the entire visual spectrum activates mRGCs, we thought that because the distribution of RGC1 and RGC2 correlated with the almost equal numbers of rods and cones, their proportions might change after exposure to light of different colors. Instead, we found that the distribution of *c-fos +* cells between RGCs sub-populations was similar, regardless of the type of light to which Stage 42 embryos were exposed for 30 minutes (Figure 6G).

Although the experiments using light colors were unsuccessful in their original intent to separate the classic photoreceptor pathway and melanopsin-driven circuits, they were nonetheless informative in several ways about the functioning of retinal circuits. First, in the central retina, fewer circuits were activated with blue light, although the proportion of activated cells in the INL and the RGC layer was comparable, suggesting that a similar type of ‘unit circuit’ was activated. Second, whereas *c-fos* expression by mRGCs was unaffected by light color (reflecting a binary readout of *c-fos* induction and/or an induction trigger by the photoreceptor pathway), TH + ACs were differentially light color-sensitive (mainly blue-responsive), indicating that at least in some cells/circuits, the triggering of *c-fos* expression is color-dependent. This latter result suggests that TH + ACs participate in a melanopsin-triggered circuit, and provide some support for a PR-activated pathway as the driver of induction of *c-fos* in mRGCs.

## Discussion

Although *c-fos* expression has been studied in the adult retina of several species [30,31], expression has been characterized only minimally in the embryonic retina [60]. Our study combined cell marker analysis with *c-fos* expression, as an indicator of neural activity, to identify at the earliest stages of retinal circuit development the specific cell types that participate in the classic IF circuit and in retinal circuits involving melanopsin-expressing cells. By this means, we mapped the retinal circuits activated by a single pulse of light, and determined the developmental stage at which they become functional.

### Wiring neuronal circuits activated by light

**Figure 7:**
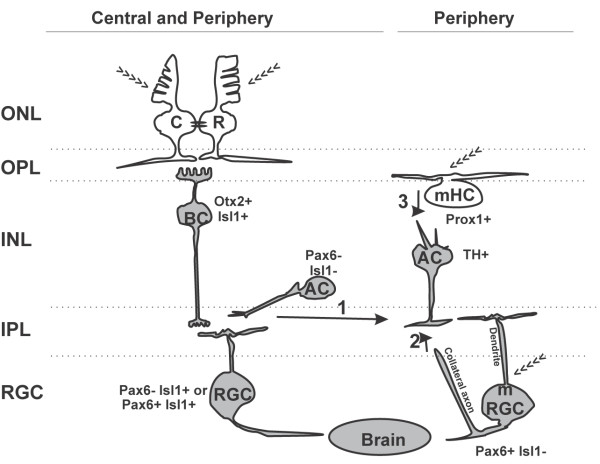
**Neuronal circuit diagram of the light input pathway in the tadpole retina.** Cells expressing *c-fos* in response to a first exposure to light are shown in gray, and we propose that they correspond to second-order or third-order neurons. Photosensitive cells (>>>) do not express *c-fos* as they are first-order neurons. Melanopsin-expressing retinal ganglion cells (mRGCs) induce *c-fos* in response to light, either by serving as first-order neurons because of their intrinsic photosensitivity, or via a role as a second-order or third-order neuron that receives synaptic inputs from other retinal cells. The classic IF circuit is present in both the central and peripheral retina. In lower vertebrates, rods (R) and cones (C) in the ONL are connected via gap junctions (black ovals) and synapse directly on a single class of On-BCs (Otx2+ / Isl1+), which drive activity in a sub-population of ACs (Pax6− / Isl1−) [59]. Finally, the pathway drives *c-fos* expression in two equally abundant RGC sub-populations (Pax6− / Isl1+ and Pax6+ / Isl1+). Not illustrated are the cells of the retinal IF circuit that do not express *c-fos* in response to light: the HCs, Off-BCs, and Pax6+ and/or Isl1+ ACs. The cells involved in non-image-forming (NIF) tasks express melanopsin and are preferentially distributed in the peripheral retina. These include the mHCs (Prox1+),mRGCs (Pax6+ / Isl1−), and dopaminergic (TH+) ACs that turn on *c-fos* with blue light. Three possible connections may induce *c-fos* expression in the TH + ACs: 1) PR-initiated inputs from On-BCs to mRGCs and/or TH + ACs; 2) synaptic interaction between an mRGC axon collateral with INL cells [62] to provide a retrograde signal from mRGCs to TH + ACs [63]; and 3) a circuit that may only exist in lower vertebrates, whereby mHCs act as first-order neurons, and interplexiform (TH+) ACs link mHCs to mRGCs.

Based on our characterization of retinal cell types expressing *c-fos* and their activation in response to a range of different wavelengths, we suggest a working model for both the classic visual network and a circuit involving melanopsin-positive cells at Stage 42 (Figure 7). At this stage, the IF circuit is newly established, the majority of RGC axons have innervated their brain targets [32-34], and the NIF circuit is functional (that is, retinal melatonin secretion is occurring) [23]. Further, the two circuits remain at least partially separate, as suggested by the differential activation of TH + cells with distinct colors of light (Figure 7).

In the vision circuit, initial light exposure activates two INL cell types downstream of the photoreceptors, the On BCs (Otx2+ / Isl1+), and a subset of ACs (Pax6− / Isl1−). Further downstream, two RGC sub-populations are activated (Pax6+ / Isl1+ and Pax6− / Isl1+). Whereas the IF circuit was distributed throughout the entire eye, we found that melanopsin-positive mRGCs and mHCs were distributed preferentially in the peripheral retina. mHCs are present in lower vertebrates [21,22,44,45,48,50,61], but are probably absent from mammals. The role of mHCs in lower vertebrates is not completely understood, although the data suggest a role in sensing ambient light [50,61]. We found that mHCs (Prox1+) were six times more abundant than mRGCs (Pax6+ / Isl1−), were localized to the outer INL, and did not induce *c-fos* in response to light, whereas the minor mRGC sub-population did induce *c-fos* in response to light. Intriguingly, we also identified a population of dopaminergic (TH+) ACs, which had similar numbers, peripheral distribution, and location to mRGCs. Further, these cells showed preferential induction of *c-fos* expression by blue light. Together, the data are suggestive of TH + cells participating in a melanopsin-triggered circuit.

An interesting component of the model is that first-order neurons do not express light-induced *c-fos,* whereas second-order and third-order neurons do. For instance, RGCs and On BCs, but not PRs, expressed *c-fos* after initial light exposure. Similarly, mHCs were found in the outer retina and did not induce *c-fos* with light exposure, suggesting that they may also serve as first-order neurons.

How TH + cells hook into the circuit is not clear, but there are three possible alternative explanations. 1) TH + cells receive inputs from On BCs via the rod/cone pathway. However, the fact that blue light was more effective than red/green light at inducing *c-fos* mRNA in TH + cells suggests some interconnection with melanopsin-positive cells (mHCs or mRGCs) or the small proportion of frog blue-sensitive cones (~10%) or rods (~3%) (Figure 7; arrow 1). 2) TH + cells receive a retrograde signal from mRGCs. A recent study revealed a population of mammalian mRGCs with collateral axons that contact cells in the INL [62]. Moreover, mRGCs induce a retrograde spike in TH + ACs [13,52,63]. Thus, the blue light-induced *c-fos* expression in *Xenopus* TH + cells might arise via an evolutionarily conserved retrograde pathway from mRGCs to TH + cells (Figure 7; arrow 2). 3) A dopaminergic AC connects mHCs to mRGCs. Interplexiform dopaminergic cells are present in teleost and frog retinas [53,64-66], but absent in mammals, such as rabbits [67]. In teleost fish, these cells extend processes to both the IPL and OPL, connecting to HCs in the OPL [64,68]. In the few cases in which we could observe the entire morphology of a TH + cell by GFP expression, small processes were observed oriented to the OPL. Thus, this circuit remains a possibility, and presumably would be wired at the same time as the synapses of PRs and BCs, as *c-fos* expression in mRGCs was also initially detected at Stage 37/38. Distinguishing between these options will depend on future experiments in which mRGCs and/or TH + cells are functionally or anatomically removed from the developing tadpole retina.

We found no difference in the ability of white, red and green light to activate *c-fos* expression in the central retina. Rod and cone opsins were sensitive across a broad range of wavelengths, so it is possible that the opsins expressed by the green-sensitive rods (97% of rods) and red-sensitive cones (86% of cones) are activated by both red and green filtered light, although it is difficult to imagine that this would happen to the same extent with the two colors. An alternative explanation, reflected in our working model (Figure 7), comes from a recent postulate regarding light-sensitive circuits in non-mammalian retinas [59], which suggests that BCs are a mixed rod-cone type, and that these cells pass on convergent signals from rods and cones via a conventional cone pathway, comprising of BC to RGC. If true in the Stage 42 *Xenopus* retina, it would explain why no differences were seen in the ratio of *c-fos +* cells in the INL and the RGC layer with different colored light.

### Wiring the classic vision IF and melanopsin-mediated circuits during development

A key motivation for our careful characterization of the classic IF and the melanopsin-mediated circuits was to understand how and when these circuits form in the developing vertebrate retina. Interestingly, the two circuits, although they mediate distinct functions, arise essentially simultaneously in the early embryo. Our results show that in *Xenopus,* retinal cells of both the classic vision and the melanopsin-pathways were functionally engaged into circuits at the same developmental time (Stage 37/38). Of course, simultaneous developmental activation in the retina does not necessarily mean concurrent functional engagement of the IF and NIF systems at the level of the brain. For instance, differences might exist in the timing of target innervation of mRGCs and the classic RGCs. Indeed, this is the case for distinct sub-populations of classic RGCs. RGCs located in the dorsal retina are first to innervate the tectum (at Stage 37/38), targeting the ventrolateral region, while the axons of RGCs located in the ventral retina innervate the dorsomedial tectum at Stage 40 [33]. We do not yet know if there are timing differences in innervation for mRGC and classic RGC axons, although our data indicate that by Stage 42, both systems activate brain regions (data not shown).

In mammals, the NIF system is functional much earlier than vision. Mammals are blind at birth, and vision is established when interconnections between retinal cells are finalized, just after the eyes open (post-natal day 12 (P12) in rodents). By contrast, the brain area that controls circadian rhythm, the SCN, is functional immediately after birth (P0) [49,69-71]. Indeed, Lupi *et al*. showed that light-induced c-fos expression in the SCN of melanopsin knock-out mice was detectable at P14 but not at P4 [71], suggesting that early SCN activation (before P10) must be driven by melanopsin, whereas activation at P14 might also involve the newly on-line rod/cone pathway. Interestingly, the NIF system is the first to become active in rodents, yet classic RGCs innervate the lateral geniculate nucleus at embryonic stages, which is much earlier than mRGC axons arrive in the post-natal SCN [2]. Most likely in *Xenopus*, non-retinal circuits also contribute to NIF tasks, given the presence of a pineal gland and melanopsin-positive brain cells. Indeed, we found (unpublished data) that *c-fos* was induced by light in the brain of Stage 42 embryos binocularly deprived prior to entry of RGC axons into the brain at Stage 32. Green *et al*. detected induction of melatonin release as a parameter of circadian activation in the pineal gland at Stage 26 (before eye formation), indicating that in lower vertebrates, as in mammals, NIF responses may also be functional before the visual system becomes active [18]. At the level of the retina, however, mRGCs and cells of the IF circuit initially express the neural activity marker *c-fos* at the same developmental time. This difference in developmental activation of the IF and NIF retinal circuits is another distinction between how the NIF system functions in *Xenopus* and mammalian retinas. Also unique to lower vertebrates is the presence of mHCs and interplexiform TH + cells, and an additional melanopsin gene (*opn4x*).

Our data provide several insights into how sensory circuits form during development, and how development differs depending on species. In *Xenopus,* the mechanism of ‘sensing’ appears to be in place well before the retinal circuits become functional; expression of the melanopsin genes was first evident at Stage 31/32, at the same time that expression commences for several opsins in presumptive PRs [72,73]. Yet, functioning of both the classic IF and mRGC-involved circuits, as determined by us by *c-fos* expression, and for the IF circuit by electrophysiological means [74], takes almost another day (Stage 37/38). Thus, in *Xenopus*, the retinal circuits by which the sensory signals are transferred to the brain become functional only as the first RGC axons contact their major IF brain target, the optic tectum. By contrast, in the mouse, a photosensing system, in the form of opsin expression by PRs, is generated post-natally, while most of the cells of the NIF circuit and *opn4m* are present embryonically [75,76]. A third pattern is seen in chick, where melanopsin genes are expressed in mRGCs (E12) prior to PR opsins, and *opn4x* is expressed specifically by mRGCs and mHCs at E15 [45]. Presumably, these differences relate to species-specific functional requirements of the melanopsin and IF circuits over the developmental period.

Additionally, our data indicate that a ‘bare-bones’ circuit comes becomes functional in the first instance, and recruits more cells over subsequent development. Certainly, many more *c-fos +* cells turn on in response to light at Stage 42 than at Stage 37/38. Moreover, we found with the IF circuit that the participating cells (On BCs, ACs, and RGCs) appeared to become active as a complete cellular unit: *c-fos* responses appeared simultaneously in all three cell types. These data raise the interesting possibility that the cellular circuit, with appropriate synaptic connections between cells, is present early, but is dormant. Activation could occur potentially by the formation of the final connections of On BCs with PRs, or by changes in the expression of ion channels that affect cellular depolarization. In this regard, rods and cones establish synapses with BCs and HCs in the INL at Stage 37/38 [55]. Continued development of the circuit then appears to occur by the addition of complete cellular units, such that the ratio of INL to RGC layer *c-fos +* cells remains constant even though the number of *c-fos +* cells continues to increase. Understanding the molecular mechanisms by which the circuit is activated and grows will be key to future studies.

## Conclusions

We characterized different cell types in the retina that were responsive (*c-fos*-expressing) to a first exposure to light and determined the developmental time at which they became active. These cell types included cells that participate in the classic IF pathway (BCs, ACs, and RGCs), and cells that in mammals mediate NIF tasks, such as mRGCs. mHCs, which were present in lower vertebrates, did not exhibit light-activated *c-fos*. All the cell types engaged in circuits at the same developmental time (Stage 37/38), and in the central retina, the cells appeared to be recruited as ‘unit circuits’ containing two to three cells in the INL per responsive cell in the RGC layer.

## Methods

### Embryos, *in vitro* fertilization, and light exposure

All procedures involving frogs and embryos were approved by the Animal Care and Use Committee, University of Calgary.

*Xenopus laevis* embryos) were obtained from chorionic gonadotrophin (Intervet Canada Ltd.) -induced egg production and *in vitro* fertilization according to standard procedures. Embryos were kept from Stage 24 onwards in dark conditions at 16°C or 22°C until the selected developmental stage for experimental analysis, primarily Stage 42. Sister embryos that were maintained in identical conditions except that they were not restricted to dark conditions, were used to stage embryos, as described by Nieuwkoop and Faber [77].

Embryos were exposed to light in a homemade chamber located in a dark room. The chamber (40 cm length × 20 cm width × 25 cm height) contained five independently powered parallel T5 bulbs (F875 cool white light fluorescent; light output, 470 lumens; color temperature, 4000; color rendering index, 60). Measurements of the luminescence in the surface of the embryo dish ranged from 800 to 6500 lux depending on the particular combination of bulbs used. If not specifically indicated, the embryos were exposed to light for 30 minutes with a combination of bulbs that generated a light intensity of 2500 lux over the dish surface. For treatments with different wavelengths, the embryos were exposed to the same sources of light passed through specific filters located over the dishes, which were engineered to allow only certain light wavelengths to enter the water. The filters used were blue, and red cut-off filters (Optosigma 077–3330 and 077–1630, respectively), and a green, filter obtained from welding goggles, whose respective scans are shown in Figure 6A. Any experimental conditions that differ are identified specifically. To avoid effects due to changes in temperature generated by light exposure, the untreated embryos were also maintained in the light chambers, but in fully covered dishes.

### RNA *in situ* hybridization

Both *opn4m and opn4x* were amplified by Reverse transcriptase-PCR from single-strand cDNA generated from whole embryos at Stage 42 using SuperScriptTM II RNase H reverse transcriptase (Invitrogen) according to the manufacturer’s instructions. PCR amplifications were carried out in a total volume of 20 μl using Fermentas PCR mix (Thermo Fisher Scientific Inc., Ottawa, Ontario). The primers used to amplify *opn4m* and *opn4x* are shown in Table 1. PCR amplification products were cloned into the pCRII-Topo vector and sequenced at the DNA Services Facility, University of Calgary. *opn4x* and *opn4m* amplified by PCR produced partial sequences with 100% sequence identity to those deposited previously in the gene bank database (BC169653 and DQ384639 respectively).

**Table 1:** Primers used for PCR

**Gene**	**Primer**	**Sequence 5′→3′**
*opn4m*	Forward	gagtctccgatctcctgcaaa
	Reverse	gccagtagggccactgtagaa
*opn4x*	Forward	gcttgcacagggagtggatac
	Reverse	actgctgggactgtcttggaa

A pCMV sport6 plasmid, containing the full-length cDNA encoding *X laevis c-fos* obtained from an image clone (Open Biosystems Image Clone number 5073759; pCMV-Sport6 *c-fos*), was used to generate *c-fos* probes. Antisense and sense riboprobes were synthesized from linearized plasmids (*Pvu*II restriction digestion for *c-fos* antisense, *Bam*H1 digestion for *opn4x* and *opn4m* antisense, and *Xho*I digestion for *opn4x* and *opn4m* sense). Riboprobes were synthesized using SP6 (sense) and T7 (antisense) RNA polymerases (Roche, Quebec, Canada), and digoxigenin (DIG)-labeled or fluorescein isothiocyanate (FITC)-labeled nucleotides (both Roche), and stored at −80°C. For slide *in situ* hybridization, embryos were fixed in MEMFA (0.1 M 3-n-morpholino propanesulfonic acid (MOPS), 2 mM ethylene glycol tetra-acetic acid (EGTA), 1 mM MgSO_4_, and 4% formaldehyde in diethylpyrocarbonate (DEPC)-treated H_2_O), and then stored in ethanol at −20°C. Cryostat sections of embryos were embedded in optimal cutting temperature (OTC) compound (Tissue-Tek), and cut into Sections 12 μm thick. Slides were incubated overnight at 65°C in hybridization buffer (10 mM NaH_2_PO_4_.H_2_O pH 7.5, 200 mM NaCl, 10 mM Tris, 50 mM EDTA, 50% formamide, 10% dextran sulfate, tRNA 1 mg/ml, and 1× Denhart’s solution (0.02% BSA, 0.02% polyvinylpyrrolidone, 0.02% Ficoll 400)) containing riboprobes, then washed, and anti-DIG antibody (1:2500; Roche) conjugated to alkaline phosphatase was added for immunodetection of the probes. The staining process used 5-bromo-4-chloro-3-indolyl phosphate (BCIP; Roche) and nitro blue tetrazolium chloride (NBT; Roche) substrates in a NTMT solution (100 mM NaCl, 100 mM Tris-Cl pH 9.5, 50 mM MgCl2, and 1% Tween 20). Proteinase K was omitted during *in situ* hybridization to allow subsequent immunohistochemistry on the same sections. For double *in situ* hybridization, DIG-labeled and FITC-labeled probes were synthesized as described above and detected sequentially by using anti-DIG (1:500 dilution) and anti-FITC (1:500 dilution) specific antibodies coupled to HRP, and the color developed with tyramide signal amplification (TSA) cyanine 3 and TSA fluorescein system kits, respectively (both Perkin Elmer, USA) in accordance with the manufacturer’s instructions. Here, and elsewhere in this study, digital images were obtained with Axiovision software, and adjusted for brightness and contrast by using Adobe Photoshop CS5.

### Immunohistochemistry post *in situ* hybridization

Immunohistochemistry was performed on sections after *in situ* hybridization using standard procedures. Briefly, the alkaline phosphatase *in situ* hybridization reaction was stopped with water, followed by PBS-T (PBS pH 7.4, 0.1% BSA (Sigma), and 0.5% Triton X100 (BDH)). Sections were blocked with blocking buffer (PBS-T plus 5% goat serum (Invitrogen) before addition of primary antibody in blocking buffer. The rabbit polyclonal antibodies Prox1 (1:400 dilution; ab37128), Otx2 (1:200; ab21990) (both Abcam), Pax6 (1:100) and Calbindin (1:200; D-28 K) (both Cedarlane), or the mouse monoclonal antibodies Islet1 (1:100; clone 394D5; DSHB, IA, USA), TH (1:100; clone LNC1) and rhodopsin (1:200 dilution; clone Rho 1D4) (both Millipore) were used. Alexa Fluor-tagged secondary antibodies (green and red; 488 and 546 nm emission, respectively; anti-mouse or anti-rabbit) diluted 1:1000 were used to detect the appropriate primary antibody. Of note, it is possible that the Otx2 antibody raised against rabbit OTX2 recognizes both Otx2 and Otx5b in *Xenopus*[38].

### Eye electroporation

Electroporation was performed as described previously [54]. A Picospritzer II was used to make repeated injections of a plasmid pCS2-GFP (0.25–1 μg/μl) through a borosilicate glass needle medial to the eye primordium of Stage 27/28 embryos. Embryos developed at room temperature until Stage 42; they were then fixed overnight in 4% paraformaldehyde, and processed for immunohistochemistry (12 μm sections) with anti-GFP (1:400 dilution, rabbit polyclonal; Invitrogen A11122) and anti-TH antibodies.

### Cell counting and analysis

For analysis of the positive cells in the central retina, digital images were captured of *c-fos* expression in single transverse central retinal sections containing the optic nerve head. If this section was in sub-optimum condition, a section just anterior or posterior was used for analysis. The number of eyes analyzed in any experiment (n) is indicated as well as the number of cells/section or total cells counted (n). Error, unless otherwise indicated, is SD. Because few cells expressed melanopsin, a minimum of eight eyes was used for melanopsin cell counts. Several sections with melanopsin-expressing cells, sometimes several from the same eye, were randomly chosen and analyzed to determine co-expression with specific markers. Cell counting was performed using the public domain ImageJ software. GraphPad Prism software (v.3.03; GraphPad Software, San Diego, CA, USA) was used for linear regression analysis and curve fitting of the kinetics of *c-fos* expression, constraining the minimum to 0 and the maximum to 100, but allowing other parameters to remain unconstrained. A time course of *c-fos* induction in the eye was analyzed semi-quantitatively by comparing the integral optical density of an image of the entire eye (×40 magnification) taken with a fixed gain and light, and expressed as a percentage between the maximum (30 minutes = 100%) and the minimum (time 0 = 0%). Statistical differences between groups were considered significant at *P* < 0.05, with Bonferroni’s correction for *post hoc* comparisons.

## Abbreviations

AC: amacrine cell; BCIP: 5-Bromo-4-chloro-3-indolyl phosphate; BC: Bipolar cell; BSA: bovine serum albumin; DEPC: Diethylpyrocarbonate; DIG: Digoxigenin; FITC: Fluorescein isothiocyanate; GFP: green fluorescent protein; HC: horizontal cell; IF: Image-forming; NIF: Non-image-forming; mHC: Melanopsin-expressing horizontal cell; MOPS: 3-n-morpholino propanesulfonic acid; mRGC: Melanopsin-expressing retinal ganglion cell; INL: Inner nuclear layer; IPL: Inner plexiform layer; ONL: Outer nuclear layer; OPL: Outer plexiform layer; OTC: Optimal cutting temperature; PBS-T: phosphate-buffered saline with Triton; Photoreceptor: PR; RGC: Retinal ganglion cell; TH: Tyrosine hydroxylase.

## Competing interests

The authors declare no competing financial interests.

## Authors’ contributions

GEB and SM designed the experiments. CLH and GEB carried out the experiments. GEB and SM analyzed and interpreted the data, and drafted the manuscript. All authors read and approved the final manuscript.
